# Mas-Related Gene (Mrg) C Activation Attenuates Bone Cancer Pain via Modulating Gi and NR2B

**DOI:** 10.1371/journal.pone.0154851

**Published:** 2016-05-06

**Authors:** Yu’e Sun, Ming Jiang, Bailing Hou, Cui’e Lu, Yishan Lei, Zhengliang Ma, Xiaoping Gu

**Affiliations:** Department of Anaesthesiology, the Affiliated Drum-Tower Hospital of Medical College of Nanjing University, Nanjing 210008, Jiangsu, China; University of Texas Medical Branch, UNITED STATES

## Abstract

**Objective:**

This study is to investigate the role of Mas-related gene (Mrg) C in the pathogenesis and treatment of bone cancer pain (BCP).

**Methods:**

BCP mouse model was established by osteosarcoma cell inoculation. Pain-related behaviors were assessed with the spontaneous lifting behavior test and mechanical allodynia test. Expression levels of MrgC, Gi, and NR2B in the spinal cord were detected with Western blot analysis and immunohistochemistry.

**Results:**

Pain-related behavior tests showed significantly increased spontaneous flinches (NSF) and decreased paw withdrawal mechanical threshold (PWMT) in mouse models of BCP. Western blot analysis showed that, compared with the control group and before modeling, all the expression levels of MrgC, Gi, and NR2B in the spinal cord of BCP mice were dramatically elevated, which were especially increased at day 7 after operation and thereafter, in a time-dependent manner. Moreover, the treatment of MrgC agonist BAM8-22 significantly up-regulated Gi and down-regulated NR2B expression levels, in the spinal cord of BCP mice, in a time-dependent manner. On the other hand, anti-MrgC significantly down-regulated Gi expression, while dramatically up-regulated NR2B expression, in the BCP mice. Similar results were obtained from the immunohistochemical detection. Importantly, BAM8-22 significantly attenuated the nociceptive behaviors in the BCP mice.

**Conclusion:**

Our results indicated the MrgC-mediated Gi and NR2B expression alterations in the BCP mice, which might contribute to the pain hypersensitivity. These findings may provide a novel strategy for the treatment of BCP in clinic.

## Introduction

Bone cancer pain (BCP) is one of the most intractable factors in patients suffering from primary bone tumors or bone metastases, which strongly influences the patients’ quality of life [1]. So far, the mechanism for BCP has not yet been fully elucidated, and the current treatments are always inevitably accompanied with significant adverse effects [2, 3]. Therefore, it is really important and urgent to develop novel efficient therapeutic strategies for BCP in clinic.

Numerous studies indicate that, in rodents, mas-related gene (Mrg) C, a sensory neuron-specific GPCR, shares substantial homogeneity with its human homolog, MrgX1, with about 45–65% amino acid sequence identity [4]. MrgC is located specifically in the small-diameter dorsal root ganglion (DRG) neurons, which are presumably nociceptive. It has been shown that, intrathecal injection of MrgC agonist, i.e., BAM8-22 and γ2-MSH, could produce antinociceptive effects and attenuate heat hyperalgesia in acute pain models [5–7]. Moreover, unpublished data from our lab has also demonstrated that, intrathecal administration of MrgC agonist BAM8-22 would attenuate BCP in a mouse model, in a dose-dependent manner.

NR2B is one of the subunits of NMDA receptor, which has been shown to be involved in the pain regulation in various injuries [8, 9]. Tyrosine phosphorylation of NR2B in the spinal cord is associated with the central sensitization, increasing dorsal the horn excitability and facilitating the sensory input, which may be responsible for BCP [10, 11]. Previous studies have demonstrated that NMDA receptor antagonists (such as ketamine) and NR2B-selective antagonists (such as ifenprodil and Ro25-6981) could exert potent analgesic effects in animal models of BCP [12, 13]. However, these antagonists have also been shown to be associated with intolerable side effects, including memory impairment and psychotomimetic effects. Therefore, it is of great importance to explore novel ways to modulate the NMDA receptors, without affecting the basal receptor activity, to achieve better analgesic effect.

In this study, the mouse model of BCP was established by the inoculation of osteosarcoma cells, and the role of MrgC in the pathogenesis and treatment of BCP was investigated. Pain-related behaviors were assessed in these mouse models, and the expression levels of Gi and NR2B in the spinal cord were also detected, before and after the manipulation of MrgC.

## Materials and Methods

### Animals and cell culture

Adult male C3H/HeJ mice, 4–6 weeks old, weighing 18–22 g, were purchased from the Vital River Experimental Animal Center, Beijing, China. These animals were habituated individually under a 12/12-h light/dark cycle, at a constant room temperature of 24°C, with free access to food and water. All the animal experimental procedures were approved by the Animal Care and Use Committee of the Affiliated Drum-Tower Hospital of Medical College of Nanjing University. Physical conditions of the mice were monitored before each behavior tests, and none of these mice died unexpectedly prior to the experimental endpoint.

Fibrosarcoma NCTC 2472 cells (2087787; American Type Culture Collection, ATCC, Manassas, VA, USA) were cultured with the NCTC 135 medium (Sigma-Aldrich, St. Louis, MO, USA) with 10% horse serum (Gibco, Grand Island, NY, USA) in a 5% CO_2_, 37°C incubator.

### Animal grouping and modeling

BCP model was established according to a previous procedure from Schwei *et al*. [14]. Briefly, mice were anesthetized with the intraperitoneal injection of 50 mg/kg pentobarbital sodium (1% in saline), and a superficial incision was made in the skin overlying the right articulatio genu. Gonarthrotomy was performed to expose the femur condyles, which were then subjected to light depression caused by dental bur. Cortex were perforated with a 30-gauge needle. For the mice in the model group (n = 60), 20 μL α-MEM containing 2×10^5^ NCTC 2472 cells were injected into the intramedullary space of the right femur using a 25-μL microsyringe. Mice in the sham group (n = 20) were injected with α-MEM alone. The injection hole was sealed with dental amalgam, and copious irrigation was implemented with saline before the wound was finally closed. All necessary measures were undertaken to minimize potential pain and distress. For example, the mice were under general anesthesia when the procedures were performed. Tourniquet was used to reduce bleeding as much as possible. Infection was prevented by operating under sterile conditions and with sterile equipments.

### Pain-related behavior tests

After modeling, the spontaneous lifting behavior test and mechanical allodynia test were performed to measure the number of spontaneous flinches (NSF) and paw withdrawal mechanical threshold (PWMT), respectively. All the tests were conducted during the light phase, and mice were allowed to acclimatize for at least 30 min before each test. NSF and PWMT were examined before animal modeling (day 0), at days 3, 5, 7, 10, and 14 after modeling, and at days 1, 3, and 7 after drug administration.

For the spontaneous lifting behavior test, a mouse was kept in an individual plexiglass compartment (10 cm × 10 cm × 15 cm) for 30 min, and the right hind limb NSF was recorded for 2 min. Lift of right hind limb not related to walking or grooming was considered as one flinch.

On the other hand, the mechanical allodynia test was conducted with the Von Frey filaments (0.16–2.0 g bending force; Stoelting, Wood Dale, IL, USA), as described by Chaplan *et al*. [15]. A mouse was placed in an individual transparent plexiglass compartment (10 cm × 10 cm × 15 cm) on a metal mesh floor with 0.5 cm × 0.5 cm squares. Filaments were oriented vertically against the plantar surface with a sufficient force causing slight bending against the animal paw, which was held for 6–8 s each time, with a 10-min interval. Positive response was defined as the brisk withdrawal or paw flinching, and the PWMT was determined using the sequentially increasing and decreasing stimulus strength (i.e., the up-and-down method). Each paw was tested five times for per stimulus strength, until the cutoff force of 2.0 g. The lowest filament strength with at least three positive responses was regarded as PWMT.

### Drug preparation and administration

Drugs were prepared and administered according to previously published protocols [5]. BAM8-22 (SML0729; Sigma-Aldrich, St. Louis, USA) and anti-MrgC antibody (orb101320; Biorbyt, San Francisco, CA, USA) were both dissolved in saline. At 14 days after modeling, mouse models were randomly divided into the following three groups: (1) the BCP model control group (n = 20), in which the tumor-bearing mice were treated with vehicle (saline) alone; (2) the BAM8-22 group (n = 20), in which the BCP mice were treated with BAM8-22; and (3) the anti-MrgC group (n = 20), in which the BCP mice were treated with anti-MrgC. For the BCP+B and BCP+A groups, 5 μL BAM8-22 (8.0 nmol) or anti-MrgC (1/20 v/v) was administered intrathecally, once per day, for 7 consecutive days [16].

### Western blot analysis

Mice were killed by decapitation after deeply anesthetized with 100 mg/kg pentobarbital sodium. Lumbar spinal cord L_3_-L_5_ segments were removed and stored in liquid nitrogen. Tissue samples were homogenized with lysis buffer, followed by centrifugation (4°C) at 13,000 rpm for 10 min. Protein concentration was determined with the BCA kit (Thermo Fisher Scientific Life Science Research, Shanghai, China). Protein sample was separated on SDS-PAGE, and then transferred onto a polyvinylidene difluoride membrane (Millipore, Billerica, MA, USA). After blocking with 5% non-fat milk at room temperature for 1 h, the membrane was incubated with rabbit anti-mouse anti-MrgC polyclonal antibody (1:500 dilution; orb101320; Biorbyt), mouse anti-mouse anti-NR2B monoclonal antibody (1:1000 dilution; ab93610; Abcam, Cambridge, MA, USA), and rabbit anti-mouse anti-Gi polyclonal antibody (1:500 dilution; ab140333; Abcam), respectively. After washing, the membrane was incubated with goat anti-rabbit IgG (1:5,000 dilution; ab6721; Abcam) or goat anti-mouse IgG (1:5,000 dilution; ab6789; Abcam). Protein band visualization was achieved with the ECL method (Santa Cruz, Santa Cruz, CA, USA), and protein quantification was performed with the IPLab software (Scanalytics, Fairfax, VA, USA). β-actin was used as reference control.

### Immunohistochemistry

For immunohistochemistry, the lumbar spinal cord L_3_-L_5_ segments were perfused with 4% paraformaldehyde in 0.1 M phosphate buffer (pH 7.4) on day 3 after drug administration. Tissues were cut into 25-μm sections on a sliding microtome. After washing with PBS, the sections were blocked with 10% (v/v) normal fetal bovine serum at room temperature for 1 h. Then the sections were incubated with rabbit anti-mouse anti-MrgC polyclonal antibody (1:250 dilution; orb101320; Biorbyt) or mouse anti-mouse anti-NR2B monoclonal antibody (1:500 dilution; ab93610; Abcam) at 4°C for 48 h. After washing, the sections were incubated with Alexa Fluor 594 goat anti-rabbit IgG (1:500 dilution; ab150084; Abcam) and Alexa Fluor 488 goat anti-mouse IgG (1:500 dilution; ab150117; Abcam) at 4°C overnight. After sealing, the sections were imaged at 200× magnification with the Leica TCS SP2 multiphoton confocal microscope (Leica Microsystems, Wetzlar, Germany), and the images were analyzed with the Image-Pro Plus software (Media Cybernetics, Rockville, MD, USA).

### Statistical analysis

Data were expressed as mean ± SD. One-way ANOVA was performed for group comparison, with LSD post hoc test when significant differences were observed. *P* < 0.05 was considered as statistically significant.

## Results

### Pain-related behaviors in BCP mice

**Fig 1:**
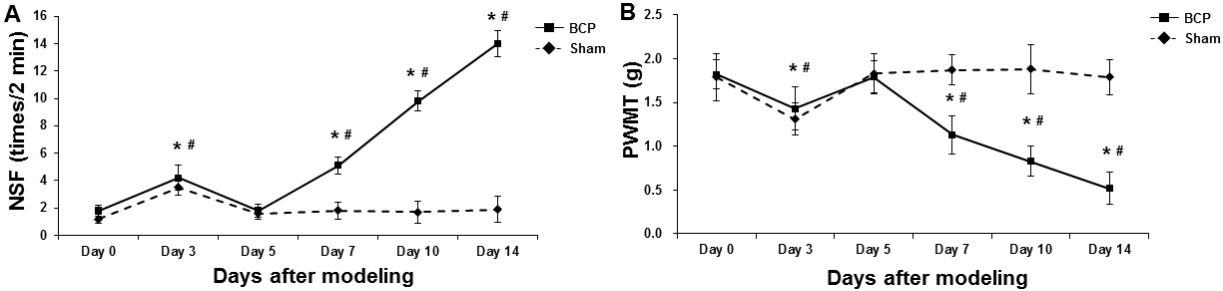
Pain-related behaviors of right hind limb in BCP mice. NSF (A) and PWMT (B) was assessed in the sham and BCP model groups (n = 8) before animal modeling (day 0), and at days 3, 5, 7, 10, and 14 after model establishment. Compared with before modeling, * *P* < 0.05; compared with the sham group at corresponding time point, ^#^ *P* < 0.05.

To assess the pain-related behaviors in the right hind limb of BCP mice, the number of spontaneous flinches (NSF) and paw withdrawal mechanical threshold (PWMT) were measured before modeling (day 0), and at days 3, 5, 7, 10, and 14 after model establishment. Our results showed that, there was no significant difference in the NSF and PWMT between the sham and model groups prior to modeling (Fig 1). For NSF, in the sham group, the NSF was significantly elevated at day 3 after operation (*P* < 0.05), which then returned to the level comparable to before modeling at day 5 after operation and thereafter (Fig 1A). On the other hand, in the model group, compared with before modeling, the NSF was also significantly elevated at day 3 after modeling (*P* < 0.05), and then slightly declined at day 5 after modeling. However, the NSF in the model group continued to increase at days 7, 10, and 14 after modeling, which was significantly higher than before modeling (all *P* < 0.05) (Fig 1A). For PWMT, in the sham group, PWMT was significantly declined at day 3 after operation (*P* < 0.05), which then returned to the comparable level with before modeling 2 days later and thereafter (Fig 1B). In the model group, compared with before modeling, the PWMT was significantly declined at day 3 after modeling (*P* < 0.05), and then slightly elevated 2 days later. However, the PWMT in the model group kept decreasing at days 7, 10, and 14 after modeling, which was significantly lower than before modeling (all *P* < 0.05) (Fig 1B). Taken together, these results demonstrate the successful establishment of mouse model of BCP, which are suitable for the following investigation.

### Up-regulated expression of MrgC, Gi, and NR2B in spinal cord of BCP mice

**Fig 2:**
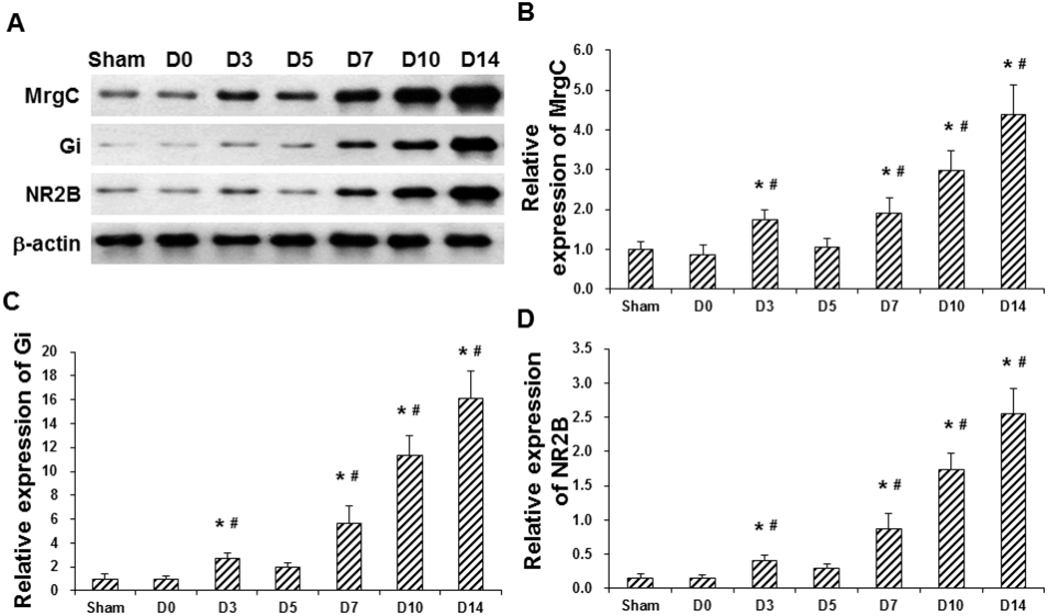
Expression levels of MrgC, Gi, and NR2B in spinal cord of BCP mice. (A) Expression levels of MrgC, Gi, and NR2B in the spinal cord of the sham mice and BCP mice were detected with Western blot analysis before animal modeling (day 0; D0), and at days 3, 5, 7, 10, and 14 (D3-D14) after model establishment. (B-D) Statistical analyses of MrgC (B), Gi (C), and NR2B (D) in the spinal cord of sham and BCP mice (n = 6). Compared with before modeling, * *P* < 0.05; compared with the sham group, ^#^ *P* < 0.05.

To investigate the expression levels of MrgC, Gi, and NR2B in the spinal cord of BCP mice, Western blot analysis was performed. Our results showed that, compared with the sham group and before modeling, all the expression levels of MrgC, Gi, and NR2B in the BCP mice were dramatically elevated after modeling, which were especially increasing at day 7 after operation and thereafter, in a time-dependent manner (all *P* < 0.05) (Fig 2). These results suggest that, the expression levels of MrgC, Gi, and NR2B in the spinal cord are up-regulated in the BCP mice, which might contribute to the pathogenesis and development of BCP.

### Effects of MrgC agonist and anti-MrgC on Gi and NR2B expression in spinal cord of BCP mice

**Fig 3:**
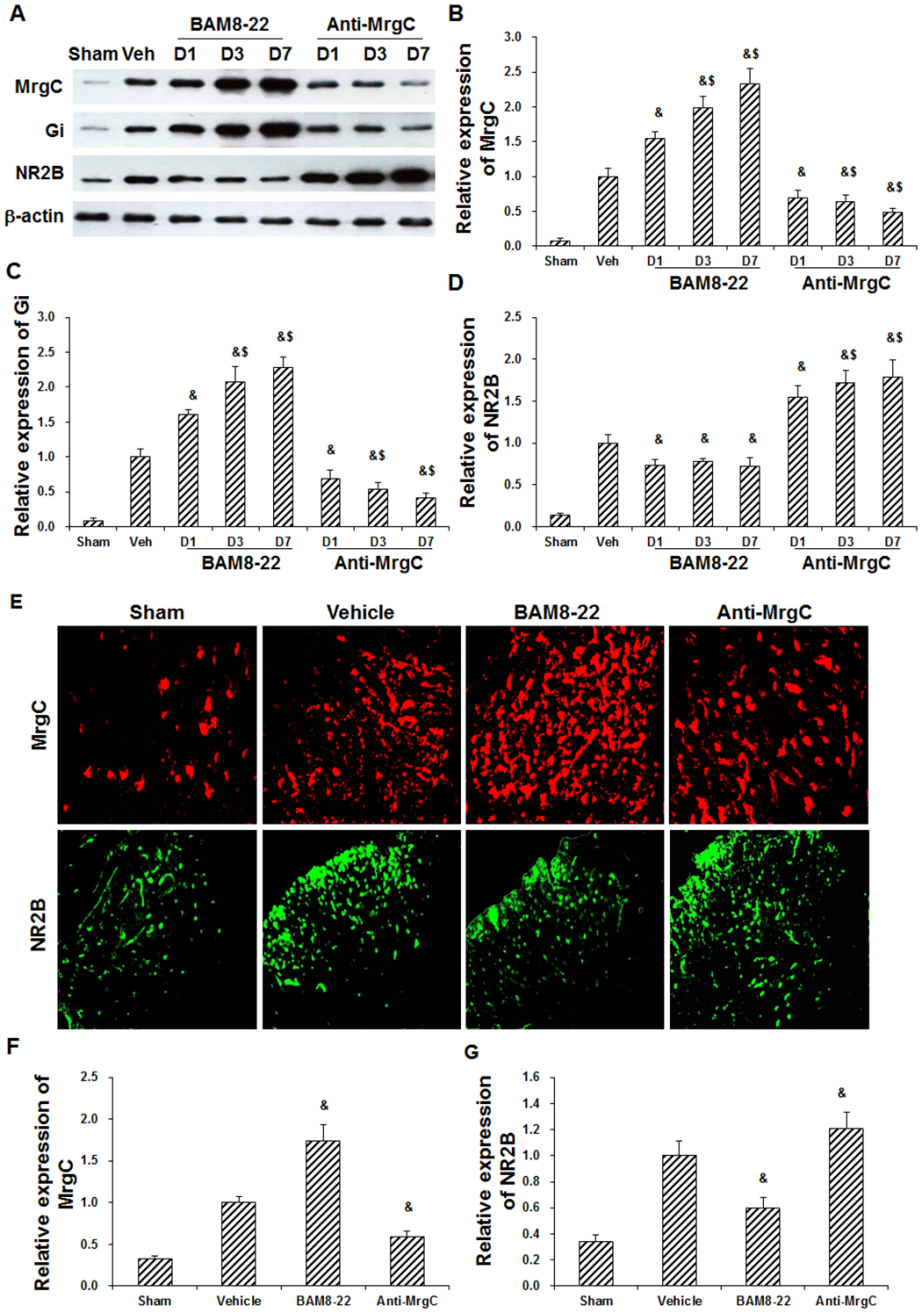
Effects of MrgC agonist and anti-MrgC on expression levels of MrgC, Gi, and NR2B in spinal cord of BCP mice. (A) The expression levels of MrgC, Gi, and NR2B in the spinal cord were detected with Western blot analysis, in sham mice, model control (Veh) mice, and model mice administered with BAM8-22 or anti-MrgC, at days 1, 3, and 7 after drug administration. (B-D) Statistical analyses of MrgC (B), Gi (C), and NR2B (D) expression levels after drug administration as indicated by Western blot analysis (n = 6). (E) Expression of MrgC (red) and NR2B (green) in the spinal cord were detected with immunohistochemistry in the sham mice, model control (vehicle) mice, and model mice treated with BAM8-22 or anti-MrgC (×200). (F-G) Statistical analyses of MrgC (F) and NR2B (G) expression levels after drug administration as indicated by immunohistochemistry (n = 6). Compared with the vehicle group, ^&^ *P* < 0.05; compared with the former time point, ^$^ *P* < 0.05.

To assess the effects of MrgC agonist and anti-MrgC on the expression levels of MrgC, Gi, and NR2B in the spinal cord of BCP mice, these animal models were first administered with BAM8-22 or anti-MrgC, and then the expression levels of MrgC, Gi, and NR2B in the spinal cord were detected with Western blot analysis and immunohistochemistry. Our results from the Western blot analysis showed that, compared with the control group (mouse models receiving vehicle), the MrgC agonist BAM8-22 significantly up-regulated the expression levels of MrgC and Gi, while significantly down-regulated the NR2B expression level, in the spinal cord of BCP mice, in a time-dependent manner (*P* < 0.05) (Fig 3A–3D). On the other hand, compared with the control (vehicle) group, the anti-MrgC significantly down-regulated the expression levels of MrgC and Gi, while dramatically up-regulated the NR2B expression level, in the spinal cord of BCP mice, in a time-dependent manner (*P* < 0.05) (Fig 3A–3D). Similar results were obtained for the immunohistochemical detection of MrgC and NR2B in the spinal cord of BCP mice. Compared with the control (vehicle) group, the expression levels of NR2B in the spinal cord were significantly declined by BAM8-22 and significantly elevated by anti-MrgC, in the BCP mice (both *P* < 0.05) (Fig 3E–3G). Taken together, these results suggest that, the manipulation of MrgC could significantly affect the expression levels of Gi and NR2B in the spinal cord of BCP mice.

### MrgC agonist attenuates nociceptive behaviors in BCP mice

**Fig 4:**
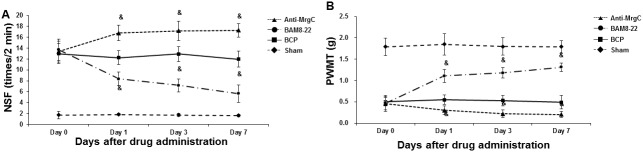
Effects of MrgC agonist and anti-MrgC on nociceptive behaviors in BCP mice. BCP mice were administered with BAM8-22 or anti-MrgC for 7 consecutive days (starting from day 14 after operation), and then the nociceptive behaviors were assessed at days 0, 1, 3, and 7 (Day 0-Day 7) after drug administration. Statistical analyses of NSF (A) and PWMT (B) in BCP mice treated with BAM8-22 or anti-MrgC. Compared with BCP model controls at corresponding time point, ^&^ *P* < 0.05.

To assess whether MrgC activation might exert analgesic effects in the BCP mice, these animal models were administered with BAM8-22 for 7 consecutive days (starting from day 14 after operation), and then the nociceptive behaviors were assessed at days 1, 3, and 7 after drug administration. Anti-MrgC was used as negative control. Our results showed that, compared with the control group (mouse models treated with vehicle), BAM8-22 significantly reduced NSF and increased PWMT in the ipsilateral hind paw of BCP mice (*P* < 0.05), in a time-dependent manner (Fig 4). On the other hand, the treatment of anti-MrgC further dramatically increased NSF and decreased PWMT in these BCP mice (*P* < 0.05), in a time-dependent manner (Fig 4). These results suggest that, the treatment of MrgC agonist could significantly attenuate the nociceptive behaviors in these BCP mice.

## Discussion

In the present study, our results showed that BCP pathogenesis was associated with the up-regulated expression of MrgC, Gi, and NR2B in the spinal cord. Moreover, the MrgC agonist BAM8-22, but not the anti-MrgC, could significantly alleviate the abnormal pain-related behaviors, through regulating Gi and NR2B expression levels. These results might provide a potential analgesic strategy for BCP in clinic.

BCP is clinically characterized by inflammation, neuropathic effects, and tumorigenic potential-[17, 18]. It has been well accepted that NMDA receptor (especially the NR2B subunit)-dependent central sensitization plays an important role in the pain hypersensitivity [19]. In the postsynaptic densities, NMDA receptors are bound to scaffolding and signaling proteins, regulating the synaptic transmission [20]. Moreover, NMDA receptor could bind to Ca^2+^/calmodulin-dependent protein kinase II (CaMKII) and members of the MAGUK family (such as PSD-95). This interaction couples the NMDA receptor-mediated Ca^2+^ influx to influence the intracellular effectors and signaling enzymes, which contribute to the NMDA receptor-dependent central sensitization [21]. Our previous study has shown that the phosphorylation of NR2B in the spinal dorsal horn in the BCP models was significantly up-regulated after fibrosarcoma cell infusion, which is accompanied by obvious pain-related behaviors [11]. Moreover, the up-regulation of NR2B phosphorylation could be significantly reduced by the intrathecal injection of MrgC agonist BAM8-22. Our results in the present study showed that BAM8-22 suppressed BCP-induced hyperalgesia, up-regulated Gi and down-regulated NR2B expression in the spinal cord, indicating that MrgC could be coupled with Gi, inducing analgesia.

Functional significance of Gi activation in the DRG neurons has been previously reported [22, 23]. Based on these findings and ours, there is the possibility that MrgC could regulate the pain sensitivity through activating the Gi signaling pathway. Of course, further studies are still needed to confirm this hypothesis. On the other hand, other studies have shown that BAM8-22 could inhibit the persistent inflammatory and chemical pain, and down-regulate the expression level of c-fos in the spinal cord [5, 6, 24]. These findings suggest that the MrgC ligands could function as anti-hyperalgesic agents. Gi protein is involved in stress and/or inflammation-induced priming [25], as well as opioid-induced hyperalgesic priming [26]. Gi activation might contribute to the antinociception of BAM8-22. BAM8-22 could dose-dependently diminish NMDA-evoked pain behaviors in rats [27], suggesting that it may induce spinal analgesia by suppressing NMDA receptor-related excitation. It has been recently reported that activated MrgC could suppress the up-regulation of pro-nociceptive mediators (e.g., nNOS and CGRP) and inhibit the c-fos expression under inflammatory pain conditions [5, 7].

In the present study, our results indicated that the MrgC activation alleviated the BCP-related behaviors, which was in line with previous findings [11, 12, 28, 29]. Implantation of tumor cells into the right femur induced progressive changes in the NSF and PWMT, indicating the successful establishment of BCP model. Meanwhile, these BCP mice were accompanied by notable increased expression levels of Gi and NR2B in the spinal cord, which might be involved in the development and maintenance of BCP. Moreover, our findings further revealed that the intrathecal administration of MrgC agonist BAM8-22 attenuated the tactile hypersensitivity and pain-related behaviors in the injured paw of these mouse models, and modulated the expression levels of Gi and NR2B in the spinal cord. However, opposite effects were observed for the anti-MrgC. These results demonstrate that MrgC, Gi, and NR2B might participate in the regulation of pain-related behaviors in these BCP mice. Further in-depth studies are still needed to explore the detailed mechanisms.

Our results showed that, the NSF were increased and the PWMT were decreased in the ipsilateral hind limb of these BCP mice, at day 7 after operation. However, the decreased PWMT at day 3 and the recovered behavior at day 5 after modeling may be attributed to the operational influences. At day 14 after operation, the BCP-related behaviors were obvious and tended to be stable. Accordingly, day 14 after operation was selected as the time point for drug intervention in these BCP mice. Considering the complexity and continuity of BCP, repetitive drug administration was applied to approximately exert the therapeutic effects and investigate the optimal dosing duration.

In conclusion, our results showed that, the expression levels of MrgC, Gi, and NR2B were dramatically elevated in the spinal cord of BCP mice. Moreover, the MrgC agonist BAM8-22 significantly up-regulated Gi and down-regulated NR2B expression levels. Importantly, BAM8-22 could attenuate the nociceptive behaviors in these BCP mice. Our results indicated MrgC-mediated Gi/NR2B expression changes in the BCP mice, which might contribute to the modulation of pain hypersensitivity. These findings may provide a novel strategy for the treatment of BCP in clinic.

## References

[1] van den Beuken-van Everdingen MH, de Rijke JM, Kessels AG, Schouten HC, van Kleef M, Patijn J. Prevalence of pain in patients with cancer: a systematic review of the past 40 years. Ann Oncol. 2007;18(9):1437–49. 10.1093/annonc/mdm056 . 17355955

[2] Boland J, Boland E, Brooks D. Importance of the correct diagnosis of opioid-induced respiratory depression in adult cancer patients and titration of naloxone. Clin Med (Lond). 2013;13(2):149–51. Epub 2013/05/18. 10.7861/clinmedicine.13-2-149 . 23681862 PMC4952630

[3] Colvin L, Fallon M. Challenges in cancer pain management—bone pain. Eur J Cancer. 2008;44(8):1083–90. Epub 2008/04/29. 10.1016/j.ejca.2008.03.001 . 18439817

[4] Zylka MJ, Dong X, Southwell AL, Anderson DJ. Atypical expansion in mice of the sensory neuron-specific Mrg G protein-coupled receptor family. Proc Natl Acad Sci U S A. 2003;100(17):10043–8. Epub 2003/08/12. 10.1073/pnas.1732949100 ; PubMed Central PMCID: PMCPmc187757. 12909716 PMC187757

[5] Jiang J, Wang D, Zhou X, Huo Y, Chen T, Hu F, et al Effect of Mas-related gene (Mrg) receptors on hyperalgesia in rats with CFA-induced inflammation via direct and indirect mechanisms. Br J Pharmacol. 2013;170(5):1027–40. 10.1111/bph.12326 23909597 PMC3949651

[6] Chen T, Cai Q, Hong Y. Intrathecal sensory neuron-specific receptor agonists bovine adrenal medulla 8–22 and (Tyr6)-gamma2-MSH-6-12 inhibit formalin-evoked nociception and neuronal Fos-like immunoreactivity in the spinal cord of the rat. Neuroscience. 2006;141(2):965–75. 10.1016/j.neuroscience.2006.04.011 . 16713112

[7] Wang D, Wang P, Jiang J, Lv Q, Zeng X, Hong Y. Activation of Mas Oncogene-Related G Protein-Coupled Receptors Inhibits Neurochemical Alterations in the Spinal Dorsal Horn and Dorsal Root Ganglia Associated with Inflammatory Pain in Rats. J Pharmacol Exp Ther. 2015;354(3):431–9. 10.1124/jpet.115.225672 . 26157044

[8] Brittain MK, Brustovetsky T, Brittain JM, Khanna R, Cummins TR, Brustovetsky N. Ifenprodil, a NR2B-selective antagonist of NMDA receptor, inhibits reverse Na+/Ca2+ exchanger in neurons. Neuropharmacology. 2012;63(6):974–82. Epub 2012/07/24. 10.1016/j.neuropharm.2012.07.012 ; PubMed Central PMCID: PMCPmc3427421. 22820271 PMC3427421

[9] Lau CG, Takeuchi K, Rodenas-Ruano A, Takayasu Y, Murphy J, Bennett MV, et al Regulation of NMDA receptor Ca2+ signalling and synaptic plasticity. Biochem Soc Trans. 2009;37(Pt 6):1369–74. Epub 2009/11/17. 10.1042/bst0371369 ; PubMed Central PMCID: PMCPmc3501105. 19909278 PMC3501105

[10] Liu Y, Cui X, Sun YE, Yang X, Ni K, Zhou Y, et al Intrathecal injection of the peptide myr-NR2B9c attenuates bone cancer pain via perturbing N-methyl-D-aspartate receptor-PSD-95 protein interactions in mice. Anesth Analg. 2014;118(6):1345–54. Epub 2014/05/21. 10.1213/ane.0000000000000202 . 24842180

[11] Gu X, Zhang J, Ma Z, Wang J, Zhou X, Jin Y, et al The role of N-methyl-D-aspartate receptor subunit NR2B in spinal cord in cancer pain. Eur J Pain. 2010;14(5):496–502. Epub 2009/10/10. 10.1016/j.ejpain.2009.09.001 . 19815434

[12] Xiaoping G, Xiaofang Z, Yaguo Z, Juan Z, Junhua W, Zhengliang M. Involvement of the spinal NMDA receptor/PKCgamma signaling pathway in the development of bone cancer pain. Brain Res. 2010;1335:83–90. Epub 2010/04/07. 10.1016/j.brainres.2010.03.083 . 20362561

[13] Stan TL, Alvarsson A, Branzell N, Sousa VC, Svenningsson P. NMDA receptor antagonists ketamine and Ro25-6981 inhibit evoked release of glutamate in vivo in the subiculum. Transl Psychiatry. 2014;4:e395 Epub 2014/06/04. 10.1038/tp.2014.39 ; PubMed Central PMCID: PMCPmc4080320. 24893066 PMC4080320

[14] Schwei MJ, Honore P, Rogers SD, Salak-Johnson JL, Finke MP, Ramnaraine ML, et al Neurochemical and cellular reorganization of the spinal cord in a murine model of bone cancer pain. J Neurosci. 1999;19(24):10886–97. Epub 1999/12/14. . 10594070 10.1523/JNEUROSCI.19-24-10886.1999 PMC6784931

[15] Chaplan SR, Bach FW, Pogrel JW, Chung JM, Yaksh TL. Quantitative assessment of tactile allodynia in the rat paw. J Neurosci Methods. 1994;53(1):55–63. Epub 1994/07/01. . 7990513 10.1016/0165-0270(94)90144-9

[16] Hylden JL, Wilcox GL. Intrathecal morphine in mice: a new technique. Eur J Pharmacol. 1980;67(2–3):313–6. Epub 1980/10/17. . 6893963 10.1016/0014-2999(80)90515-4

[17] Mercadante S, Fulfaro F. Management of painful bone metastases. Curr Opin Oncol. 2007;19(4):308–14. Epub 2007/06/05. 10.1097/CCO.0b013e3281214400 . 17545792

[18] Pan HL, Zhang YQ, Zhao ZQ. Involvement of lysophosphatidic acid in bone cancer pain by potentiation of TRPV1 via PKCepsilon pathway in dorsal root ganglion neurons. Mol Pain. 2010;6:85 Epub 2010/12/02. 10.1186/1744-8069-6-85 ; PubMed Central PMCID: PMCPmc3004845. 21118579 PMC3004845

[19] Campbell JN, Meyer RA. Mechanisms of neuropathic pain. Neuron. 2006;52(1):77–92. Epub 2006/10/04. 10.1016/j.neuron.2006.09.021 ; PubMed Central PMCID: PMCPmc1810425. 17015228 PMC1810425

[20] D'Mello R, Marchand F, Pezet S, McMahon SB, Dickenson AH. Perturbing PSD-95 interactions with NR2B-subtype receptors attenuates spinal nociceptive plasticity and neuropathic pain. Mol Ther. 2011;19(10):1780–92. Epub 2011/03/24. 10.1038/mt.2011.42 ; PubMed Central PMCID: PMCPmc3188755. 21427709 PMC3188755

[21] Doucet MV, Levine H, Dev KK, Harkin A. Small-molecule inhibitors at the PSD-95/nNOS interface have antidepressant-like properties in mice. Neuropsychopharmacology. 2013;38(8):1575–84. Epub 2013/03/01. 10.1038/npp.2013.57 ; PubMed Central PMCID: PMCPmc3682152. 23446451 PMC3682152

[22] Malin SA, Molliver DC. Gi- and Gq-coupled ADP (P2Y) receptors act in opposition to modulate nociceptive signaling and inflammatory pain behavior. Mol Pain. 2010;6:21 Epub 2010/04/20. 10.1186/1744-8069-6-21 ; PubMed Central PMCID: PMCPmc2865444. 20398327 PMC2865444

[23] Gu XY, Liu BL, Zang KK, Yang L, Xu H, Pan HL, et al Dexmedetomidine inhibits Tetrodotoxin-resistant Nav1.8 sodium channel activity through Gi/o-dependent pathway in rat dorsal root ganglion neurons. Mol Brain. 2015;8:15 Epub 2015/03/13. 10.1186/s13041-015-0105-2 ; PubMed Central PMCID: PMCPmc4350947. 25761941 PMC4350947

[24] Cai Q, Jiang J, Chen T, Hong Y. Sensory neuron-specific receptor agonist BAM8-22 inhibits the development and expression of tolerance to morphine in rats. Behav Brain Res. 2007;178(1):154–9. Epub 2007/01/18. 10.1016/j.bbr.2006.12.014 . 17227682

[25] Dina OA, Khasar SG, Gear RW, Levine JD. Activation of Gi induces mechanical hyperalgesia poststress or inflammation. Neuroscience. 2009;160(2):501–7. Epub 2009/03/12. 10.1016/j.neuroscience.2009.03.001 ; PubMed Central PMCID: PMCPmc2673984. 19275929 PMC2673984

[26] Bianchi E, Galeotti N, Menicacci C, Ghelardini C. Contribution of G inhibitory protein alpha subunits in paradoxical hyperalgesia elicited by exceedingly low doses of morphine in mice. Life Sci. 2011;89(25–26):918–25. Epub 2011/10/20. 10.1016/j.lfs.2011.09.025 . 22008476

[27] Chen T, Hu Z, Quirion R, Hong Y. Modulation of NMDA receptors by intrathecal administration of the sensory neuron-specific receptor agonist BAM8-22. Neuropharmacology. 2008;54(5):796–803. Epub 2008/02/06. 10.1016/j.neuropharm.2007.12.010 . 18249418

[28] Qu XX, Cai J, Li MJ, Chi YN, Liao FF, Liu FY, et al Role of the spinal cord NR2B-containing NMDA receptors in the development of neuropathic pain. Exp Neurol. 2009;215(2):298–307. Epub 2008/12/03. 10.1016/j.expneurol.2008.10.018 . 19046970

[29] Ren BX, Gu XP, Zheng YG, Liu CL, Wang D, Sun YE, et al Intrathecal injection of metabotropic glutamate receptor subtype 3 and 5 agonist/antagonist attenuates bone cancer pain by inhibition of spinal astrocyte activation in a mouse model. Anesthesiology. 2012;116(1):122–32. Epub 2011/11/30. 10.1097/ALN.0b013e31823de68d . 22123524

